# Evaluation of the Environmental DNA Method for Estimating Distribution and Biomass of Submerged Aquatic Plants

**DOI:** 10.1371/journal.pone.0156217

**Published:** 2016-06-15

**Authors:** Saeko Matsuhashi, Hideyuki Doi, Ayaka Fujiwara, Sonoko Watanabe, Toshifumi Minamoto

**Affiliations:** 1 Graduate School of Simulation Studies, University of Hyogo, Minatojima-minami-machi, Chuo-ku, Kobe, Japan; 2 Graduate School of Human Development and Environment, Kobe University, Tsurukabuto, Nada-ku, Kobe, Japan; 3 Graduate School for International Development and Cooperation, Hiroshima University, Kagamiyama, Higashi-Hiroshima, Japan; Central Michigan University, UNITED STATES

## Abstract

The environmental DNA (eDNA) method has increasingly been recognized as a powerful tool for monitoring aquatic animal species; however, its application for monitoring aquatic plants is limited. To evaluate eDNA analysis for estimating the distribution of aquatic plants, we compared its estimated distributions with eDNA analysis, visual observation, and past distribution records for the submerged species *Hydrilla verticillata*. Moreover, we conducted aquarium experiments using *H*. *verticillata* and *Egeria densa* and analyzed the relationships between eDNA concentrations and plant biomass to investigate the potential for biomass estimation. The occurrences estimated by eDNA analysis closely corresponded to past distribution records, and eDNA detections were more frequent than visual observations, indicating that the method is potentially more sensitive. The results of the aquarium experiments showed a positive relationship between plant biomass and eDNA concentration; however, the relationship was not always significant. The eDNA concentration peaked within three days of the start of the experiment in most cases, suggesting that plants do not release constant amounts of DNA. These results showed that eDNA analysis can be used for distribution surveys, and has the potential to estimate the biomass of aquatic plants.

## Introduction

Freshwater ecosystems provide resources and habitats for many species [1]; however, these habitats have been severely damaged by human activities, such as land-use change, hydrological modification, climate change, and biological invasions [2,3]. The biodiversity of freshwater habitats is declining faster than that of terrestrial ecosystems [4–7], and therefore, it is necessary to efficiently monitor and assess the changing biodiversity status for their effective management and conservation. Species distributions and biomass are fundamental for understanding ecosystem and biodiversity status; however, these are difficult to estimate accurately in aquatic environments.

Recently, the environmental DNA (eDNA) method for the direct detection of species-specific DNA from water has been recognized as a powerful tool for monitoring aquatic species [8,9]. This method can be used in freshwater ecosystem surveys to (i) detect the distribution of species and (ii) estimate species biomass and/or abundance. The eDNA method has been applied to detect the distribution of several animals such as fish [10,11], amphibians [12,13], reptiles [14,15], mammals [16,17], and crustaceans [18]. It has also been used to estimate biomass and/or abundance of species experimentally and practically in several animal species, including common carp [19–21], Rocky Mountain tailed frog [22], Idaho giant salamander [22], common spadefoot toad [16], and great crested newt [16]. In these survey methods, only 0.015–10 L of water is needed for a sample. Therefore, eDNA analysis could reduce sampling costs, time, and labor [23], and be used to efficiently investigate species distributions and abundance/biomass in extensive regions.

Although eDNA methods have been developed for animal species, they have not been used extensively for monitoring aquatic plants. Scriver *et al*. (2015) [24] showed that the DNA of ten aquatic plant species could be detected from experimental aquarium samples, and Fujiwara *et al*. (2016) [25] demonstrated that eDNA of a submerged species, *Egeria densa*, could be detected from natural ponds. These reports showed that eDNA analysis had a potential use in field surveys for aquatic plants; however, more research should be carried out prior to its application for aquatic plant management. It is generally not easy to observe and identify small populations of submerged plants in the field, so eDNA methods are expected to increase research efficiency.

In the present study, we examined the use of eDNA analysis to estimate species occurrence and biomass of submerged aquatic plant species. To examine the use for detecting distribution, we conducted field surveys for the submerged species *Hydrilla verticillata*, whose distribution is decreasing across Japan. We compared its estimated occurrence by using eDNA analysis, visual observation, and past distribution records. To examine the utility of eDNA method for estimating biomass, we conducted aquarium experiments using two submerged plant species, *H*. *verticillata* and *E*. *densa*, to determine the relationships between the species’ eDNA concentrations and their biomass. From the results of these investigations, we assessed the application of the eDNA method to practical distribution surveys and estimations of plant biomass.

## Materials and Methods

### Study species

*H*. *verticillata* (Hydrocharitaceae) is a submerged aquatic plant native to Asia and Australia [26,27]; however, in Japan its distribution has recently become limited. In eastern Japan, it is threatened with local extinction (according to the Local Red Data Books of Tochigi, Ishikawa, and Nagano Prefectures). However, it has expanded its distribution as an invasive species in North America, South America, New Zealand, Africa, and Europe [28,29].

*E*. *densa* is a submerged aquatic plant native to South America. The species has invaded in North America, Europe, and Asia [30]. It was introduced to Japan in the 1920s [31] and became a common aquatic plant in southwestern Japan in 1980s [32]. Recently, *E*. *densa* populations have been observed in many rivers and ponds and have influenced native plants [32]. eDNA detection has been used to successfully detect this species in several ponds [25].

### Development of a primers/probe set for *H*. *verticillata*

To detect *H*. *verticillata* DNA by real-time PCR, we developed an *H*. *verticillata*-specific primers/probe set. We obtained *matK* sequences for *H*. *verticillata* and seven related species distributed in Japan (*Blyxa echinosperma*, *Blyxa japonica*, *E*. *densa*, *Elodea nuttalli*, *Hydrocharis dubia*, *Ottelia alismoides*, and *Vallisneria asiatica*) from the National Center for Biotechnology Information database. By comparing the *H*. *verticillata* sequence with those of the seven related species, we designed primers and a probe for *H*. *verticillata* (S1 Fig) using Primer Express 3.0 (Life Technologies, Carlsbad, CA, USA). We selected primers that had two or one species-specific nucleotide site(s) within five bases of the 3’-ends of the forward and reverse primers, respectively, because the 3’ end of the primers is important for specificity [33]. To determine the specificity of the primers/probe set, we performed real-time PCR using DNA extracted from the leaf tissue of *H*. *verticillata* and *E*. *densa* and examined whether amplicon of *H*. *verticillata* was confirmed but that of *E*. *densa* was not.

### Field survey to estimate the species distribution

**Fig 1:**
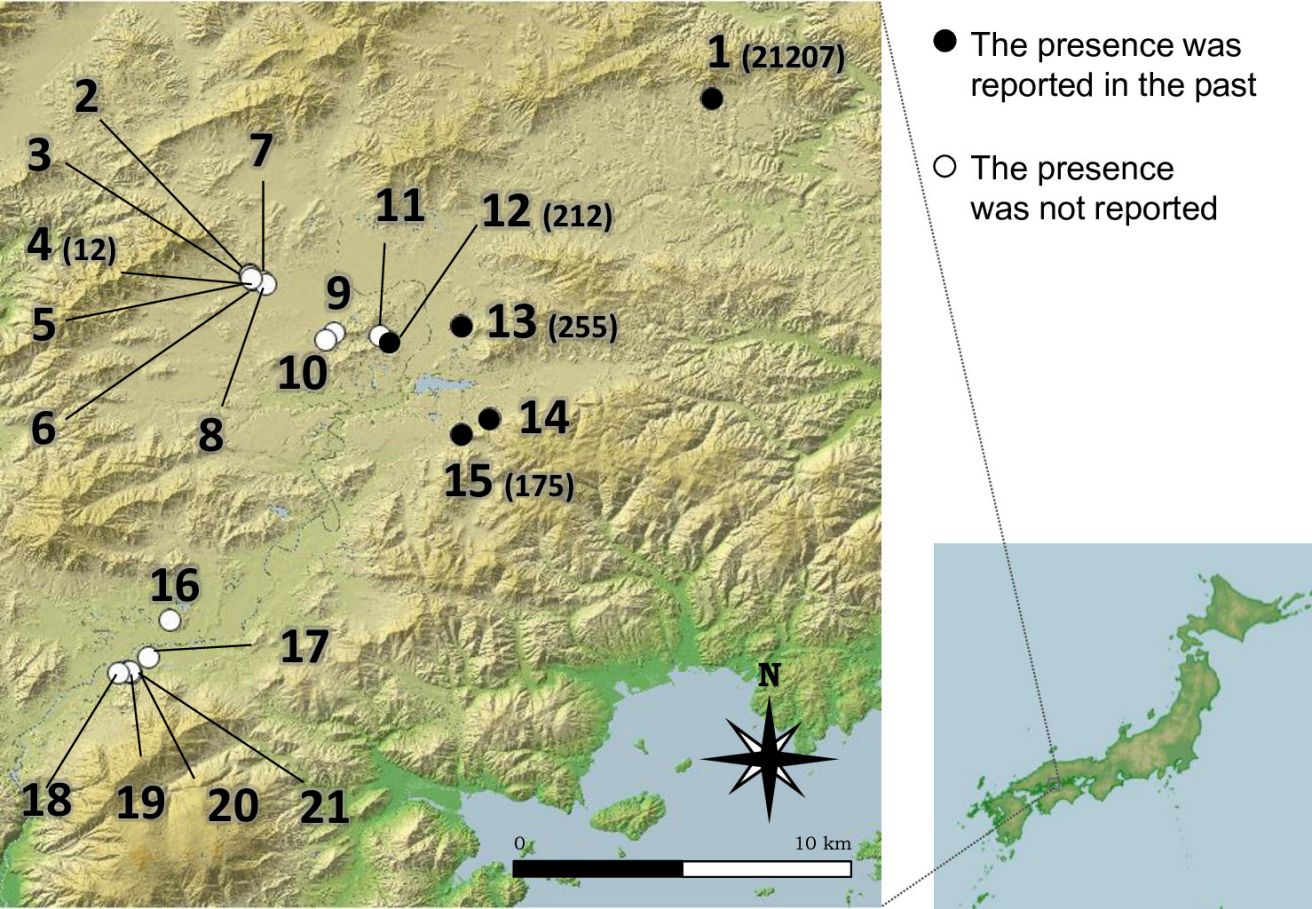
The location of 21 ponds in the field survey. The numbers correspond to the site IDs in Table 1. Closed and open circles indicate that the presence of *Hydrilla verticillata* that was or was not reported in the past, respectively. The number in parentheses shows the eDNA concentration (copies L^-1^). This map was constructed using Fundamental Geospatial Data published by the Geographical Survey Institute, Japan.

We compared the distribution of *H*. *verticillata* through three methods: estimations from the eDNA analysis, visual observation, and past records from 21 ponds in Higashi-Hiroshima City, Japan (Fig 1, Table 1). We conducted the survey from 11 June to 14 October 2014 because it was easier to find this species during summer and autumn. The ponds were selected for their accessibility. In five of the 21 ponds, *H*. *verticillata* plants were observed between 1999 and 2002 [34] (Table 1). We collected a single 1 L water sample for eDNA analysis from the surface within 3 m from the shore of each pond. The sampling point at each pond was selected randomly. The target species was observed at two ponds (Pond 1 and 12). We visually recognized the target species from the sampling point of Pond 1 where it was dominant, but did not from the sampling point of Pond 12. The plastic sampling bottles were treated with a 0.06% sodium hypochlorite solution before sampling to avoid contamination. The samples were immediately placed in a cool box until they were filtered. We also recorded the presence/absence of *H*. *verticillata* based on visual observations from the shore following the method of Takahara *et al*. 2013 [11]. A person observed *H*. *verticillata* in the water while walking along the shoreline for 10–20 min depending on the shoreline length. We also used the rake toss method at some sites where we could not observe submerged plants clearly due to water turbidity or other reasons. When *H*. *verticillata* was observed, we collected samples of leaf tissue. No specific permits were required for the described field studies.

**Table 1:** Sampling locations, information from past distribution records, and results of the field surveys. Sites where the rake toss method was used are shown with “○”. For the past distribution and visual observation from this study, “+” indicates that *Hydrilla verticillata* was confirmed visually, and “–” indicates that it was not. eDNA detection shows the number of positives in eight PCR replicates. An asterisk indicates a site where eDNA detection was checked and confirmed in the next year. The eDNA concentration at each site was estimated by quantitative real-time PCR.

Site ID	Latitude (N)	Longitude (E)	Surface area (m^2^)	Rake toss	Past record	Visual observation	eDNA detection	eDNA concentration (copies L^-1^)
1	34.4734	132.8239	83		+	+	8/8*	21207
2	34.4249	132.6962	2121	○	−	−	0/8	Not tested
3	34.4243	132.6961	1510	○	−	−	0/8	Not tested
4	34.4236	132.6965	1021	○	−	−	3/8*	12
5	34.4234	132.6971	491	○	−	−	0/8	Not tested
6	34.4231	132.6969	1963		−	−	0/8	Not tested
7	34.4226	132.6997	3393	○	−	−	0/8	Not tested
8	34.4222	132.7006	1454	○	−	−	0/8	Not tested
9	34.4088	132.7196	3206	○	−	−	0/8	Not tested
10	34.4068	132.7172	3503	○	−	−	0/8	Not tested
11	34.4077	132.7323	6065	○	−	−	0/8	Not tested
12	34.4062	132.7345	998	○	+	+	8/8*	212
13	34.4108	132.7549	1521	○	+	−	8/8*	255
14	34.3850	132.7625	2820	○	+	−	0/8	Not tested
15	34.3806	132.7549	6017	○	+	−	8/8*	175
16	34.3292	132.6740	2526		−	−	0/8	Not tested
17	34.3190	132.6683	2101		−	−	0/8	Not tested
18	34.3144	132.6599	872	○	−	−	0/8	Not tested
19	34.3147	132.6626	2857		−	−	0/8	Not tested
20	34.3149	132.6630	981		−	−	0/8	Not tested
21	34.3145	132.6633	872		−	−	0/8	Not tested

To confirm the results of eDNA detection at the ponds where we detected the eDNA, we conducted water sampling and the eDNA analysis again in the next year. On 10th July 2015, we collected 1 L water from all the ponds where the eDNA was detected (Ponds 1, 4, 12, 13, and 15).

Each water sample was filtered using two GF/F glass filters (mesh size: ~0.7 μm, GE Healthcare Japan, Tokyo, Japan) within 6 h of sampling to capture eDNA. The amount of filtered water varied (0.6–1 L) according to the timing when each filter became clogged. All filtration equipment was bleached and rinsed with DNA-free pure water between rounds of filtration to prevent cross-contamination. To determine whether contamination among samples occurred during filtration, 1 L of DNA-free distilled water was filtered after the filtration of the samples on each sampling day as a negative control. The filters were stored at –20°C until DNA extraction. The captured DNA was collected from each filter by a centrifuge by using a Salivette tube (Sarstedt, Nümbrecht, Germany) and extracted with a DNeasy Blood & Tissue Kit (Qiagen, Hilden, Germany) following the method of Uchii *et al*. (2016) [35]. DNA was eluted in 100 μL of Buffer AE and stored at –20°C until PCR was conducted.

To confirm identification, we also extracted DNA from the collected leaf tissue using a DNeasy Blood & Tissue Kit on a different day to avoid contamination. For each sample, 1–2 leaves were added to 200 μL of TE and ground with a pestle in a 1.5 mL tube. Then 200 μL of Buffer AL and 20 μL of Proteinase K were added and the sample was incubated at 56°C for 30 min. After incubation, DNA was extracted following the standard protocol of the kit.

Real-time PCR was performed with the StepOnePlus Real-Time PCR System (Life Technologies) using the designed primers/probe set (see Results). Each TaqMan reaction contained 900 nM each primer, 125 nM TaqMan probe, 10 μL of PCR master mix (TaqMan Environmental Master Mix 2.0; Life Technologies), and 3 μL of the DNA solution, for a final volume of 20 μL. The PCR conditions were as follows: 10 min at 95°C, and 55 cycles of 15 s at 95°C, and 1 min at 58°C. Non-quantitative real-time PCR (without a size standard) was performed in eight replicates for each sample to screen for *H*. *verticillata*. For the samples that detected the target DNA in at least one of the eight replicates, real-time quantitative PCR was performed in triplicate with the same conditions mentioned above except for the addition of a quantification standard, and the mean of the three was treated as the concentration of each sample. To prepare standard DNA for real-time PCR, the target sequence of the amplification was inserted into a pMD20-T vector (Takara, Shiga, Japan), and the vector was digested with EcoRI. A standard curve was constructed using 30,000, 3,000, 300, 30, and 3 copies of the standard DNA per PCR reaction. If the target DNA was not detected in a well, the concentration value of the well was assigned a zero [19]. All PCR plates contained negative controls of ultrapure water in place of template DNA: eight tubes for detection assays and three for quantification assays, respectively.

To confirm the primer specificity, the PCR amplicons that were positive were directly sequenced after treatment with ExoSAP-IT (USB Corporation, Cleveland, OH, USA). Sequences were determined by a commercial sequencing service (Eurofins Genomics Tokyo, Tokyo, Japan).

We calculated Cohen’s Kappa value to test the correspondence of the ponds where *H*. *verticillata* was reported in the past to those where it was detected by visual observation or by eDNA. Cohen’s Kappa value of 1 implies a perfect match and values less than 1 imply a less-than-perfect match. The analyses were conducted using the function ‘kappa2’ of the package ‘irr’ in R version 3.1.2 [36].

DNA was extracted from the water samples collected in the following year (2015), and the target DNA detection was checked with the same method mentioned above except that the number of PCR replicates was three.

### Aquarium experiments

We conducted aquarium experiments to evaluate the relationships between the eDNA concentrations and the biomass of aquatic plants. Before the start of the experiments, *H*. *verticillata* and *E*. *densa*, which were purchased from an aquarium shop for the experiments, were grown in an incubator at 20°C with an 18:6 h light/dark cycle for more than one week. We confirmed the survivorship and growth of the 1 cm plant fragments under these conditions. We cut their stems into 1 or 4 cm lengths after removing the apical meristem, and put each fragment in a clear plastic bag (120 × 85 mm) filled with 250 mL of aged tap water. The next day one 1 cm-, one 4 cm-, and two 4 cm-fragments were each placed in separate clear plastic bags (340 × 240 mm) filled with 2 L of aged tap water and 0.5 mL of the fertilizer Hyponex (N:P:K = 6%:10%:5%. Hyponex, Osaka, Japan) was added to make three conditions with low, middle, and high biomass. Each condition had six replicates.

To examine the effect on the eDNA detection of other species that grew in the same place, we developed three two-species conditions. In summary, the three conditions consisted of *H*. *verticillata* and *E*. *densa* as follows: a 1 cm-fragment of *H*. *verticillata* and two 4 cm-fragments of *E*. *densa*, a 4 cm-fragment of *H*. *verticillata* and of *E*. *densa*, and two 4 cm-fragments of *H*. *verticillata* and a 1 cm-fragment of *E*. *densa* (S2 Fig). All conditions were treated in the same way as mentioned above. Four replicates were set for each two-species condition. For a negative control, we prepared a plastic bag with 2 L of aged tap water and nutrition, but without plant fragments. A total of 49 bags, i.e., 32 bags of single-species conditions (6 replicates × 3 conditions × 2 species), 12 bags of two-species conditions (4 replicates × 3 conditions), and 1 negative control were placed in clear plastic containers and arranged randomly in an incubator (NKsystem, Osaka, Japan) at 20°C with a 18:6 h light/dark cycle. The arrangement was changed every few days to reduce positional bias in the incubator. The photosynthetic photon flux density (PPFD) in the incubator was ca. 130 μmol photons m^-2^ s^-1^. The light source consisted of metal halide lamps.

On 1, 2, 3, 5, 7 and 10 d after the start of the experiment, we collected 15 mL of water in 50 mL plastic centrifuge tubes from each water bag. For homogenous distribution of the eDNA concentration, the water was sampled after mixing by pipetting. Immediately after sampling, 1.5 mL of 3 mol L^-1^ sodium acetate (pH 5.2) and 33 mL of absolute ethanol were added to each water sample, and the tubes were stored at –20°C until DNA extraction [37]. The tube was centrifuged at 10,000 × *g* for 1 h at 4°C to precipitate eDNA. The eDNA pellet was eluted with 100 μL of TE (pH 8.0). The DNA solution was added to 20 μL of proteinase K and incubated at 60°C for 1 h. After heating at 95°C for 10 min, the samples were stored at –20°C until PCR analysis. The DNA extractions and PCR were carried out in different rooms to avoid contamination. After the last sampling, each plant fragment was dried in an oven at 60°C for two days and weighed to obtain its biomass.

Real-time quantitative PCR was performed in triplicate, and the mean value was used for the analyses. Amplification was performed using the previously mentioned conditions. eDNA of *E*. *densa* was amplified using the following primers/probe set: forward primer 5’-CATTTCTCCTTCATTGTATTCTTTCACA-3’, reverse primer 5’-ATTTCTATCTGTATCGTAGCCACCAA-3’, and TaqMan probe 5’-FAM-CGGGTCCGAACAGAAATGCTTCTCTCT-TAMRA-3’ [25]. We used a pUC57 plasmid, to which 373 bp of the *trnL*–*trnF* intergenic spacer region including the target sequence was inserted, as a quantification standard of *E*. *densa*. A standard curve of both species was constructed using 30,000, 3,000, 300, 30, and 3 copies per PCR reaction.

We tested whether the eDNA concentration was correlated with the biomass by using a generalized linear mixed model (GLMM) with a Gaussian distribution and identity link using the function ‘lme’ of the package ‘nlme’ in R version 3.1.2. The eDNA concentration was treated as the response variable of GLMM, the biomass and the number of days counted from the start of the experiment were treated as the explanatory variable, and the individual bag was treated as the random effect.

## Results

### Primers/probe set design and specificity

We designed a primers/probe set for *H*. *verticillata* using selected *matK* sequences (forward primer, 5’- TTTGCGCGAATATGTAGAACTTGT-3’; reverse primer, 5’- GCCAAGGTTTTAGCACAGGAAA-3’; TaqMan MGB probe, 5’- FAM-ATTATTGTAGTGGATCTTCA–NFQ–MGB-3’). We confirmed that this primers/probe set amplified DNA extracted from *H*. *vertillata* tissue but not *E*. *densa* tissue, and the DNA sequence of the amplicon was confirmed by direct sequencing.

### Estimate of the distribution by eDNA analyses and visual observation

We observed *H*. *verticillata* visually in two of 21 ponds (Ponds 1 and 12, Fig 1, Table 1). This species was dominant in Pond 1, but sparse in Pond 12. We did not get any detections/observations from the rake toss trial. The Cohen’s kappa value between the visual observation and the past distribution was 0.504.

We detected eDNA of this species in five ponds (Ponds 1, 4, 12, 13, and 15), including the two ponds where we observed plants. For the samples from four ponds (Pond 1, 12, 13, and 15), the target eDNA was detected in all eight replicates of real-time PCR, while three of eight replicates were positive for Pond 4 (Table 1). By resampling in the following year, we confirmed that the target DNA was detected from the water samples collected at the five ponds; the detection rate in Ponds 1, 12, 13, and 15 was 3/3 PCR replicates, and that in Pond 4 was 1/3 PCR replicates. All sequences of PCR amplicons from eDNA samples and DNA that was extracted from the leaf tissue corresponded to that of *H*. *verticillata*. Four (Ponds 1, 12, 13, and 15) of the five eDNA-positive ponds corresponded to the ponds where *H*. *verticillata* has been observed in the past records, and in one pond (Pond 4), *H*. *verticillata* had not been observed in the past survey (Fig 1, Table 1). The Cohen’s kappa value between the eDNA analysis and the past distribution was 0.728, closer to 1 (perfect match) than that between the visual observation and the past distribution.

The concentration of *H*. *verticillata* eDNA in Pond 1, where the species was dominant, was the highest (21,207 ± 2055 copies L^-1^), and that in Pond 4 was the lowest (12 ± 21 copies L^-1^) among the five ponds (Table 1). The concentrations in the other ponds ranged from 175 to 255 copies L^-1^. For this quantification assay, the standard curve (slope: −3.37, y-intercept: 41.003) had R^2^ = 0.992, and the PCR efficiency was 99.4%.

### Relationships between eDNA concentrations and biomass

**Fig 2:**
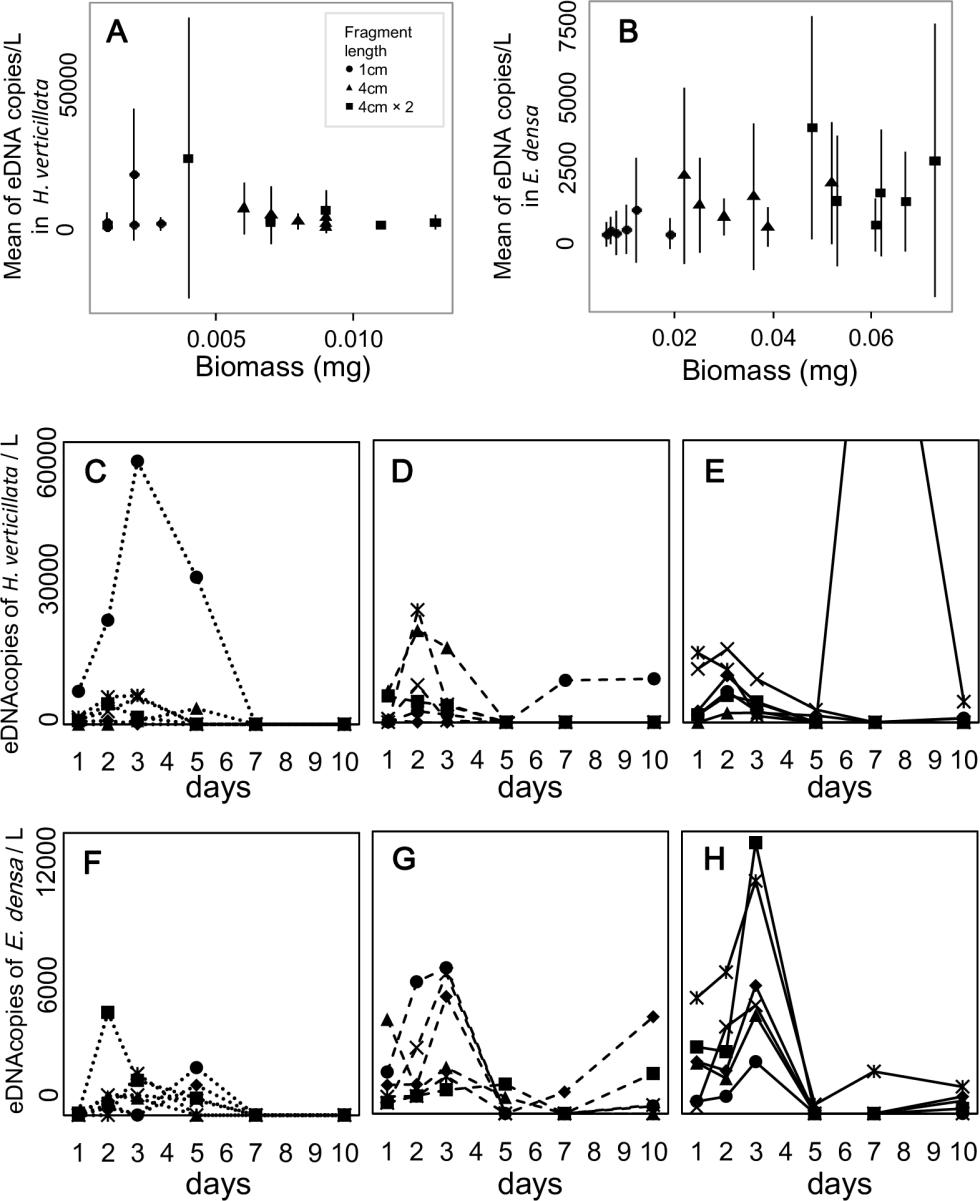
The relation between eDNA concentration and biomass, and the temporal changes of eDNA concentration in *Hydrilla verticillata* and *Egeria densa* under single-species conditions. The means of eDNA concentration were calculated for each water bag (A, B). The error bars indicate ±1 standard deviation. Dotted lines (C, F), dashed lines (D, G), and solid lines (E, H) show the three conditions with low, middle, and high biomass, respectively. Six symbols in each graph indicate the six replicates for each condition. Note that biomass of some samples with a 4 cm-fragment is larger than those with two 4 cm-fragments.

**Fig 3:**
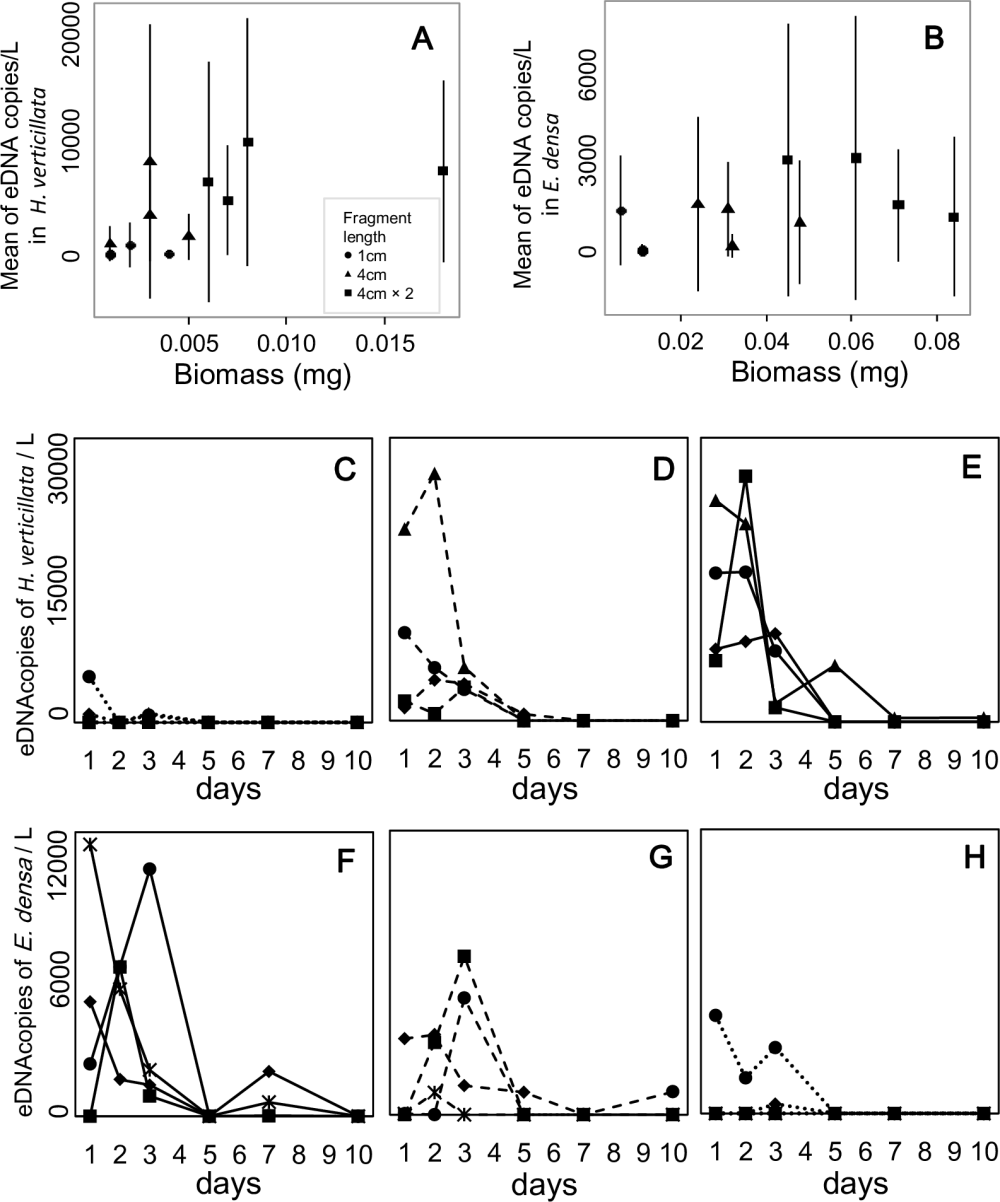
The relationship between eDNA concentration and biomass, and the temporal changes of eDNA concentration in *Hydrilla verticillata* and *Egeria densa* under two-species conditions. The mean eDNA concentrations were calculated for each water bag (A, B). The error bars indicate ±1 standard deviation. Dotted lines (C,H), dashed lines (D, G), and solid lines (E, F) show the three conditions with low, middle, and high biomass, respectively. Six symbols in each graph indicate the six replicates for each condition.

The relationships between eDNA concentration and plant biomass were significantly positive for *E*. *densa* in single-species conditions (Table 2, Figs 2 and 3). On the other hand, the relationship was not significant for *H*. *verticillata* in single-species conditions. In two-species conditions, it was marginally significant (*P* < 0.1) for both species.

**Table 2:** Results of generalized linear mixed models analyzing the effects of biomass and the number of experimental days on the eDNA concentration in the aquarium experiments for *Hydrilla verticillata* and *Egeria densa*. The results of random effect were shown in S2 Table.

			Coefficient	SE	*t* value	*P*
**A**	*H*. *verticillata*	Biomass	-89.684	135.210	-0.663	0.517
**Single-species**		Days	-0.281	0.092	-3.040	0.003
**condition**	*E*. *densa*	Biomass	11.239	4.288	2.621	0.019
		Days	-0.097	0.026	-3.763	< 0.001
**B**	*H*. *verticillata*	Biomass	172.023	84.492	2.036	0.072
**Two-species**		Days	-0.420	0.085	-4.939	< 0.001
**condition**	*E*. *densa*	Biomass	9.729	5.337	1.824	0.098
		Days	-0.138	0.036	-3.794	< 0.001

The eDNA concentrations peaked within 3 d of the start of the experiment in 85% of the experimental bags. The effect of the sampling date on the eDNA concentrations was significant in the *E*. *densa* kept in the single-species condition and in both species in two-species conditions. In *H*. *verticillata*, the effect was not significant (Table 2); however, 83% of the bags also had their concentration peaks within 3 d of initiation. eDNA was not detected in all samples. The mean sampling day for undetected samples per water bag was 2.72 ± 1.02 (45.3 ± 17.0%; *N* = 18) for *H*. *verticillata* and 2.33 ± 1.28 (38.8 ± 21.3%; *N* = 18) for *E*. *densa* under single-species conditions, and 3.17 ± 1.70 (52.8 ± 28.3%; *N* = 12) and 3.17 ± 1.70 (52.8 ± 28.3%; *N* = 12) under two-species conditions, respectively. In particular, the non-detection rate increased in the latter half of the experiment (5 d, 7 d, and 10 d). However, some bags with low biomass under two-species conditions did not have detected eDNA throughout the experiment; the number of such bags was one and two per four bags in *H*. *verticillata* and *E*. *densa*, respectively.

For all PCR reactions in the *H*. *verticillata* and *E*. *densa* quantification assays, the range of standard curve R^2^ values was 0.984–0.995 and 0.988–0.995, and that of PCR efficiencies was 92.8–106.4% and 91.5–107.6%, respectively. No negative control samples were positive for the target DNA.

## Discussion

In the present study, we developed a specific primers/probe set for *H*. *verticillata* and established a species detection method using eDNA analysis. We successfully showed that eDNA analysis has great potential for determining the distribution of submerged aquatic plants. The use of eDNA to estimate plant biomass was suggested in the aquarium experiment using *H*. *verticillata* and *E*. *densa*. In addition, the experimental results highlighted factors that could influence plant eDNA concentrations and also some issues that need to be addressed.

### Use of eDNA analysis in detecting distributions

The results of our field investigation demonstrated that eDNA analysis could be used for distribution detection of the submerged aquatic plant, *H*. *verticillata*. We detected *H*. *verticillata* in three of 19 ponds where we did not observe the species visually and two of two ponds where we did. The occurrences detected by eDNA analysis corresponded closely to the past distribution record. These results suggested that it is difficult to accurately detect distributions of submerged aquatic plants by a single visual observation, but a single eDNA analysis could provide more information about distributions of aquatic plants.

Although we detected the eDNA but failed to observe plants at Ponds 4, 13 and 15 in the 2014 survey, we confirmed the detection of *H*. *verticillata* DNA again in the following year. In addition, we could visually observe the target species at Pond 15 in August 2015 (Saeko Matsuhashi *et al*., personal observation). From these supporting experiments and observations, we concluded that the positive signals in Ponds 4, 13, and 15 were not false positives.

Although Pond 14 had a record of *H*. *verticillata* from 1999 to 2002, its eDNA was not detected in our survey in 2014. In Higashi-Hiroshima City, where the field sites were located, *H*. *verticillata* is not regarded as an endangered species even though its distribution recently decreased; Sonoko Watanabe *et al*. (not published) confirmed the change in its distribution. In 1999 and 2000, they conducted field research at 10 ponds, where this species was observed between 1980 and 1981 [38], and did not observe this species in seven of the 10 ponds (Sonoko Watanabe *et al*. personal observation). Therefore, *H*. *verticillata* might have disappeared in Pond 14 within the last few decades. In contrast, we detected target eDNA in Pond 4, where the species has not been previously reported. There are two possibilities for this mismatch. One is that the species has been introduced to this pond recently, thus it was not observed in 1999–2002. The other possibility is that conventional observation methods cannot detect this species in ponds owing to difficulty in surveying the whole water surface, or plant dormancy, or the small population size. Since the concentration of eDNA in this pond was only 12 copies L^-1^, this low concentration could be indicative of a small population. Although we cannot clarify why this species had not been reported in the past record, our study demonstrates that eDNA surveys can find new occurrences of aquatic plants.

It was reported that eDNA analysis of aquatic plant species was as accurate in detection as visual observations of *E*. *densa* [25]. The present study showed that eDNA analysis was more sensitive than visual observations in detecting *H*. *verticillata* occurrences; the species has been decreasing recently in Japan while increasing in its nonnative range such as the U.S. [39]. This suggests that eDNA analysis could contribute to the conservation and management of submerged aquatic plant species such as *H*. *verticillata*.

### Use of eDNA analysis to estimate biomass

Although the scale of the aquarium experiment was much smaller than that of a natural population, the results provided basic information on the relationship between eDNA concentration and plant biomass, and showed that eDNA analysis could potentially be used to determine the biomass of aquatic plants. In addition, the results suggested that other factors could influence eDNA concentration. The positive relationship between plant biomass and eDNA concentration was detected in *E*. *densa*. However, the effects of biomass were sometimes not significant. Since the differences among replicates in eDNA concentrations could make the significance unclear, it is necessary to examine what factors contribute to individual differences.

The eDNA concentration changed temporally; it fluctuated during the experiment but peaked within 3 d of the start of the experiment. Although we cannot clarify the mechanism of the peak formation in this study, similar peaks were observed in other studies [19,25] and may be caused by stress from experimental conditions. It is possible that this hypothesis could apply to the present study.

While monitoring temporal changes in eDNA concentration during the experiment, we found that eDNA was occasionally not detected. This provided two important suggestions. First, it is most likely that eDNA of both species can be degraded within a few days under experimental conditions (20°C with an 18:6 h light/dark cycle). For example, we found that the eDNA concentrations decreased from more than 1,000 copies L^-1^ to 0 copies L^-1^ in one or two days in many experimental water bags. This implies that it was possible that most of the eDNA was degraded within only one or two days. This has been observed in animal species, where eDNA degradation proceeded in a short period [40,41]. The high degradation speed should allow us to determine the state of a population in real time and to avoid the misdetection of DNA from species that were historically present. Second, it was suggested that plants may not always release detectable quantities of DNA (Figs 2 and 3). Although previous studies of aquarium experiments using animals reported an eDNA concentration increase during the experiment or the maintenance of a low level after the peak [16,19,40–43], we could not find cases showing a change from high to zero in a short time period as shown in Figs 2 and 3. Plants and animals differ in as cell and tissue structures, cell functions, and metabolic systems, so the difference in the change of eDNA concentration may be attributable to individual mechanisms that release eDNA. Clarifying how eDNA is released from a plant is important to collect eDNA more efficiently.

The rate of non-detected eDNA increased in the two-species condition. Particularly, in the 1 cm fragments that were added to a bag with two 4 cm fragments of the other species, the rate of non-detected eDNA was higher than that in the other conditions. This result suggests that the amount of released eDNA or the detection rate decreased depending on the habitat water quality and/or the environment or the amount of PCR inhibitors such as polysaccharides. To address this hypothesis, the relationship between the amount of released eDNA and water quality must be examined.

### Perspective

The present study demonstrated the potential use of eDNA analysis to estimate occurrence and biomass in submerged aquatic plants. To apply eDNA analysis to field surveys for the management and conservation of aquatic plant populations, the accuracy of the analysis should be improved to avoid risks of misestimates in species distribution, abundance, and biomass. To address the accuracy, preceding studies of animal eDNA can provide useful insights. For example, water condition, environmental factors, growth process, and the distance between a water sampling point and the location of a target population may influence eDNA concentrations [40,44]. In this study, we selected the sampling points randomly, however, the sampling strategy effect should be examined in a future study. Estimates of particle sizes in released eDNA could be important regarding the origin, state, and fate of the eDNA [45,46]. These approaches should be applied to aquatic plants to refine plant eDNA methods and contribute to understanding the state of natural plant populations.

## Supporting Information

S1 Fig Specificity of a designed PCR primers/probe set for *Hydrilla verticillata* in *matK* and sequence information of *H*. *verticillata* and seven related species in *matK*. The gray-colored parts indicate *H*. *verticillata*-specific sites.
(PDF)

S2 Fig Schematic drawings of the aquarium experiments. Single-species conditions (A) were set up both for *H*. *verticillata* and *E*. *densa*.
(PDF)

S1 Table MIQE checklist. (XLS)

S2 Table Results of the random effects of generalized linear mixed models analyzing the effects of biomass and the number of experimental days on the eDNA concentration in the aquarium experiments for *Hydrilla verticillata* and *Egeria densa*. (DOCX)
