# Supplementary material for: Evaluation of the Environmental DNA Method for Estimating Distribution and Biomass of Submerged Aquatic Plants
Source: PLoS One. 2016 Jun 15;11(6):e0156217. doi: 10.1371/journal.pone.0156217 (PMC4909283; doi:10.1371/journal.pone.0156217)
Supplement: S1 Fig — The gray-colored parts indicate H. verticillata-specific sites. (PDF) [file pone.0156217.s001.pdf]

## Figure S1

| | |
|---|---|
| *Blyxa echinosperma* | ---GATTTGGTCGGATATGTAGAAACCTTTC--- |
| *Blyxa japonica* | ---GATTTGGTCGGATATGTAGAAACCTTTC--- |
| *Egeria densa* | ---GATTTGGTCGGATATGTAGAAATCTTTC--- |
| *Elodea nuttallii* | ---GATTTGGTCGGATATGTAGAAATCTTTC--- |
| *Hydrocharis dubia* | ---GATTTGGTCGGATATGTAGAAATCTTTC--- |
| *Ottelia alismoides* | ---GATTTTGTCGGATATGTAGAAATCTTTC--- |
| *Vallisneria asiatica* | ---GATTTGGTCGGATATGTAGAAATTTTTC--- |
| *Hydrilla verticillata* | ---AATTTGCGCGAATATGTAGAACTTGTTC--- |
| Forward primer | TTTGCGCGAATATGTAGAACTTGT |

| | |
|---|---|
| *Blyxa echinosperma* | ---TCATTATTATAGTGGATCCTCAAA--- |
| *Blyxa japonica* | ---TCATTATTATAGTGGATCCTCAAA--- |
| *Egeria densa* | ---TCATTATTATAGTGGATCCTCAAA--- |
| *Elodea nuttallii* | ---TCATTATTATAGTGGATCCTCAAA--- |
| *Hydrocharis dubia* | ---GCATTATTATAGCGGATCCTCAAA--- |
| *Ottelia alismoides* | ---TCATTATTATAGTGGATCCTCAAA--- |
| *Vallisneria asiatica* | ---TCATTATTATAGTGGATCTTCAAA--- |
| *Hydrilla verticillata* | ---TCATTATTGTAGTGGATCTTCAAA--- |
| Probe | ATTATTGTAGTGGATCTTCA |

| | |
|---|---|
| *Blyxa echinosperma* | ---ACTTTCATGTGCTAGAACCTTAGCTC--- |
| *Blyxa japonica* | ---ACTTTCATGTGCTAGAACCTTGGCTC--- |
| *Egeria densa* | ---ACTTTCATGTGCTAGAACCTTGGCTC--- |
| *Elodea nuttallii* | ---ACTTTCATGTGCTAGAACCTTGGCTC--- |
| *Hydrocharis dubia* | ---ATTTTCATGTGCTAGAACCTTGGCTC--- |
| *Ottelia alismoides* | ---ACTTTCATGTGCTAGAACCTTGGCTC--- |
| *Vallisneria asiatica* | ---ACTTTCATGTGCTAGAACCTTGGCTC--- |
| *Hydrilla verticillata* | ---ACTTTCCTGTGCTAAAACCTTGGCTC--- |
| Reverse primer (Reverse complemented) | TTTCCTGTGCTAAAACCTTGGC |
