# Supplementary figures and images for: Evaluation of the Environmental DNA Method for Estimating Distribution and Biomass of Submerged Aquatic Plants
Source: PLoS One. 2016 Jun 15;11(6):e0156217. doi: 10.1371/journal.pone.0156217 (PMC4909283; doi:10.1371/journal.pone.0156217)

Figure S2

(A) Single-species conditions

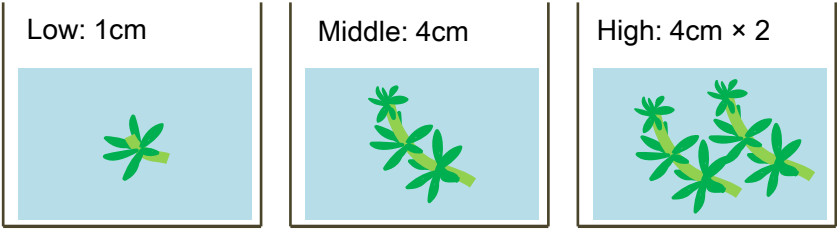

(B) Two-species conditions

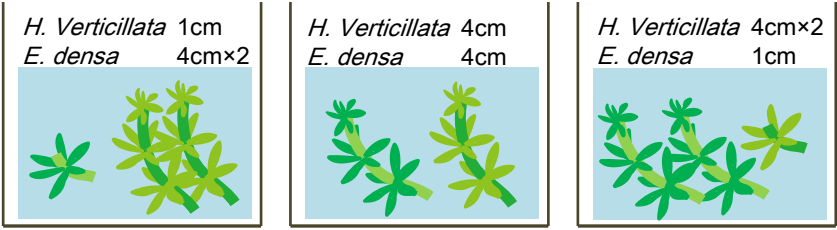

Supplement: S2 Fig — Single-species conditions (A) were set up both for H. verticillata and E. densa. (PDF) [file pone.0156217.s002.pdf]
