# Supplementary material for: Evaluation of the Environmental DNA Method for Estimating Distribution and Biomass of Submerged Aquatic Plants
Source: PLoS One. 2016 Jun 15;11(6):e0156217. doi: 10.1371/journal.pone.0156217 (PMC4909283; doi:10.1371/journal.pone.0156217)
Supplement: S2 Table — (DOCX) [file pone.0156217.s004.docx]

**Table S2**

|  |  | Intercept | Residual |
| --- | --- | --- | --- |
| Single-species condition | *H. verticillata* | 1.726 | 2.887 |
|  | *E. densa* | 0.209 | 0.831 |
| Two-species condition | *H. verticillata* | 1.004 | 2.134 |
|  | *E. densa* | 0.239 | 0.951 |
